# Automated echocardiography view classification and quality assessment with recognition of unknown views

**DOI:** 10.1117/1.JMI.11.5.054002

**Published:** 2024-08-30

**Authors:** Gino E. Jansen, Bob D. de Vos, Mitchel A. Molenaar, Mark J. Schuuring, Berto J. Bouma, Ivana Išgum

**Affiliations:** a Amsterdam University Medical Center, Department of Biomedical Engineering & Physics, Amsterdam, The Netherlands; b University of Amsterdam, Informatics Institute, Amsterdam, The Netherlands; c Amsterdam University Medical Center, Department of Cardiology, Amsterdam, The Netherlands; d Amsterdam University Medical Center, Department of Radiology & Nuclear Medicine, Amsterdam, The Netherlands

**Keywords:** echocardiography, view classification, image quality assessment, open set recognition, cardiology

## Abstract

**Purpose:**

Interpreting echocardiographic exams requires substantial manual interaction as videos lack scan-plane information and have inconsistent image quality, ranging from clinically relevant to unrecognizable. Thus, a manual prerequisite step for analysis is to select the appropriate views that showcase both the target anatomy and optimal image quality. To automate this selection process, we present a method for automatic classification of routine views, recognition of unknown views, and quality assessment of detected views.

**Approach:**

We train a neural network for view classification and employ the logit activations from the neural network for unknown view recognition. Subsequently, we train a linear regression algorithm that uses feature embeddings from the neural network to predict view quality scores. We evaluate the method on a clinical test set of 2466 echocardiography videos with expert-annotated view labels and a subset of 438 videos with expert-rated view quality scores. A second observer annotated a subset of 894 videos, including all quality-rated videos.

**Results:**

The proposed method achieved an accuracy of 84.9%±0.67 for the joint objective of routine view classification and unknown view recognition, whereas a second observer reached an accuracy of 87.6%. For view quality assessment, the method achieved a Spearman’s rank correlation coefficient of 0.71, whereas a second observer reached a correlation coefficient of 0.62.

**Conclusion:**

The proposed method approaches expert-level performance, enabling fully automatic selection of the most appropriate views for manual or automatic downstream analysis.

## 1 Introduction

Transthoracic echocardiography (TTE) is an essential part of the cardiac patient pathway and is characterized by speed of acquisition, high temporal resolution, cost-efficiency, and low patient burden. An exam may contain ∼10 to 50 clipped videos (cine loops) of standardized scan planes (views) showing a cross-section of the heart, typically with multiple videos of the same scan plane.

Despite a multitude of semi-automated tools provided by (commercially) available software packages, echocardiographic interpretation requires a fair amount of manual interaction and analysis.^1^^,^^2^ Semi-automatic analysis tools require specific views of the heart and thus require the end-user to manually select the right videos. Besides selecting the appropriate view, it is crucial for sonographers to identify the video with the highest image quality as multiple videos of the same view are often acquired. These additional steps motivate the need to integrate both automatic view classification and quality assessment into the clinical workflow. Such integration would streamline the process and improve current semi-automatic analysis tools.

The goal of view classification in echocardiography is to automatically infer the view of the heart, which is defined by the acoustic window through which the patient is scanned, and the cross-sectional plane that is acquired. For the past two decades, this problem has been addressed with machine learning (ML) methods. As Penatti et al.^3^ outlined, early ML approaches for view classification discriminated different views based on preprocessed image features. For example, Ebadollahi et al.^4^ applied an algorithm to detect and model the cardiac chambers as a graph structure and then used a support-vector machine to classify the view. Later, with the advent of deep neural networks (DNNs) and deep convolutional neural networks (CNNs) in particular, the focus shifted to end-to-end learning. Gao et al.^5^ proposed a two-stream CNN to classify the view in echocardiography videos. The method incorporated cardiac motion information by processing regular image data in one stream while concurrently processing optical flow images in the other. Madani et al.^6^ employed a convolutional neural network for view classification and obtained saliency maps to address the interpretability of inferred predictions. To better align with the requirements of downstream tasks, Zhang et al.^7^ included more fine-grained apical classes in their classification model and added a separate class for outliers. Østvik et al.^8^ employed a 2D CNN to detect the optimal scan plane in a 3D volume acquired by 3D echocardiography. Howard et al.^9^ evaluated a number of architectural approaches with CNNs and found that a two-stream network similar to Gao et al.’s^5^ method led to the best view classification performance, which approached the inter-observer level of agreement. The most recent efforts focused on minimizing computational cost while upholding state-of-the-art performance. For example, Vaseli et al.^10^ proposed a knowledge distillation technique. Azarmehr et al.^11^ proposed a neural architecture search for view classification. This resulted in an efficient architecture of half a million parameters that outperformed a computationally heavy DenseNet201^12^ with 20 million parameters on the dataset described by Howard et al.^9^

Although existing echocardiography view classification methods achieve close to expert-level performance with deep neural networks, they have been evaluated on curated (i.e., closed) datasets. For example, Howard et al.^9^ excluded 4% of their data because they were considered unclassifiable either because the view changed during a video or because the video lacked visible anatomical landmarks. On the other hand, Østvik et al.^8^ addressed the detection of unknown data by assigning an output logit to the class, but to evaluate the method, they used a nonclinical dataset. Zhang et al.^7^ trained their model with a clinical dataset including outliers but did not evaluate the model’s response to novel, truly unseen classes of data that were not included in the training set.

The problem of unclassifiable data is greatly exacerbated in real-time clinical deployment. Training data typically consist of retrospectively collected videos that the sonographer recorded only after diligently positioning the ultrasound probe to obtain the optimal view. Therefore, while transitioning between optimized views, the sonographer exposes the view classification algorithm to unknown categories of data, potentially leading to incorrect view classification.

The recognition of unknown data can be viewed as an open set recognition problem.^13^ Open set recognition addresses the inability of machine learning methods to recognize data that are very different from the training data: the open set of unknown data. By definition, open set recognition has a twofold goal: To classify samples that belong to the known set of classes from the training distribution and to recognize samples that are unknown (i.e., being out-of-distribution). In open set recognition, out-of-distribution detection is not viewed in isolation.^14^^,^^15^

In deep neural networks (DNNs), recognition of unknown data is typically performed using an anomaly detection score such as the maximum Softmax probability or maximum logit activation.^16^ In addition, several other scores have been proposed, such as the Mahalanobis distance metric,^17^ OpenMax,^18^ and more advanced approaches such as one based on generative modeling.^19^ However, Vaze et al.^15^ and Dietterich and Guyer^20^ demonstrated that the simple logit- or Softmax-based approach still achieves state-of-the-art performance on important computer vision benchmarks.

The problem of detecting unknown views can be valuable in addressing automatic quality assessment in echocardiography. In poor-quality echocardiograms, the view is typically unrecognizable because cardiac structures are either poorly visible or simply missing. Such missing features strongly correlate with reduced image quality. As the output of DNN classifiers can be interpreted as a weighted sum of familiar features,^20^ a DNN view classifier is expected to assign low output activations to a poor-quality echocardiogram. Zhang et al.^7^ therefore proposed to use the maximum Softmax probability from their view classifier as a measure of quality, but they found only modest associations between quality and this measure.

Even when addressed manually by experts, image quality assessment is a challenging task in echocardiography: it is time-consuming and subjective, thus prone to large inter- and intra-rater variability.^21^ This apparent subjectivity exists in spite of standardized scoring protocols, such as the one developed by Gaudet et al.,^22^ who created questionnaires to systematically rate the quality of each view. The questions inquire about the presence of structures, as well as appropriate gain, imaging depth, centering, and axial alignment with the ultrasound beam. Abdi et al.^23^^,^^24^ employed this protocol to define a reference standard for automatic view quality assessment with deep neural networks. Similarly, Labs et al.^25^ designed their own quality assessment questionnaire to define a reference standard for training their DNN. Although Labs et al.^25^ only evaluated apical two- and four-chamber views, their questionnaire inquired about more detailed features than Gaudet et al.’s^22^, with questions about left-ventricle foreshortedness and chamber clarity. Furthermore, Liao et al. ^21^ proposed to model the uncertainty in automatic quality assessment as their reference-standard quality ratings showed high intra-rater variability. Zamzmi et al.^26^ combined the task of view classification and quality assessment using a single neural network backbone with two classification heads: one for view classification and one for image quality assessment.

**Fig. 1:**
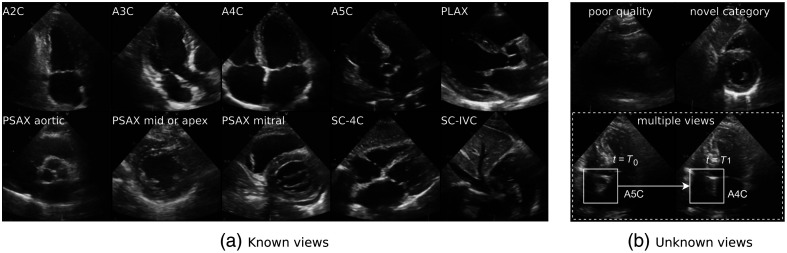
Transthoracic echocardiography view classes. (a) The known classes as part of the training set—Top row: apical two-, three-, four-, and five-chamber view; the parasternal long axis view (PLAX). Bottom row: parasternal short axis (PSAX) at the aortic valve level; PSAX at the mid-ventricle or apex; PSAX at the mitral valve; subcostal four-chamber view; the subcostal view with inferior vena cava. (b) The unknown classes to be detected while excluded from the training set—Top row: poor image quality and a novel category (here: subcostal short-axis). Bottom row: multiple views in one video.

To address the challenges of automatic view classification, unknown view detection, and quality assessment, we propose an approach where we utilize a single deep learning network to classify echocardiography views, detect unknown data, and assess view quality. The network discriminates 10 commonly analyzed views: four parasternal views, four apical views, and two subcostal views [Fig. 1(a)]. In addition, we leverage the maximum logit activations from the network to detect unknown echocardiography views, namely: (i) Videos with poor image quality, (ii) videos from a novel category, and (iii) videos fluctuating between multiple views. Finally, we employ the feature embeddings of the trained neural network to predict a view quality score with a linear classifier, which is trained separately.

This paper is organized as follows: Sec. 2 describes our dataset, Sec. 3 outlines the proposed method, Sec. 4 describes the relevant metrics for the evaluation, and Sec. 5 describes the experiments and their results. Additional experiments are described in Sec. 6, where we compare the method with a different approach to anomaly detection, and we evaluate our view classification network on the public CAMUS^27^ and Echonet-LVH^28^ datasets. Finally, we provide a discussion and conclusion in Secs. 7 and 8, respectively. For experimental comparisons regarding anomaly scores and model architectures, we refer to the Secs. 9.1 and 9.2 in the Appendix, respectively.

## 2 Data

### 2.1 Data Collection

We included a set of 619 retrospectively collected transthoracic echocardiography studies, comprising 5366 videos, derived from 598 unique patients following approval by the Amsterdam UMC Medical Research Ethics Committee. Among these, 378 studies involving 357 patients were collected from Amsterdam UMC—location University of Amsterdam (Center A), whereas 241 studies from 241 patients were collected from the Amsterdam UMC—location Free University (Center B).^29^ The dataset is further detailed in Table 1. Details about training and test data division and the resulting view-label statistics will be provided in Sec. 5.1.

**Table 1:** Details of collected echocardiography studies.

	Center A (University of Amsterdam)	Center B (Free University)
Studies	378	241
Patients	357	241
% Male	59%	59%
Age—years, median (IQR)	67 (15.7)	65 (15.0)
Manufacturer	GE	Philips
Machine types (studies)	Vivid: E9 (201), E95 (28), q (18), 7 (44), S6 (86), S70 (1)	EPIQ 7C (232), Affiniti 70C (9)
Frame rate—Hz, median (IQR)	55 (7.0)	50 (6.5)
Frames, median (IQR)	108 (37)	99 (36)
Heart cycles, median (IQR)	2.1 (0.1)	2.0 (0.1)

The included patients presented symptoms of angina pectoris (chest pain) or dyspnea (shortness of breath) at the time of their first echo exam, with most patients being suspected of chronic coronary syndromes. The Amsterdam UMC Medical Research Ethics Committee waived the need for informed consent.

### 2.2 Reference Annotations

To define a reference standard for view classification, two expert cardiologists assigned one of the following 10 view labels to each video: apical two-, three-, four-, or five-chamber view; parasternal long-axis view (PLAX); parasternal short-axis view (PSAX) at the level of the aortic valve, mitral valve, or mid/apex; the subcostal four-chamber view; or the subcostal view of the internal vena cava [Fig. 1(a)]. If a video did not unambiguously fit into any view class, it was labeled as one of three unknowns, i.e., open or out-of-distribution categories: novel category, corresponding to an identifiable view category absent in the training set; a poor quality video, indicating a view unidentifiable by an expert; and multiple views, indicating that the video fluctuates between two or more known classes within a single video sequence [Fig. 1(b)].

To define a reference standard for the quality of the videos, a subset of the data was rated based on a view quality questionnaire described by Gaudet et al.^22^ The questionnaire contains ordered multiple-choice questions, which evaluate the visibility of the imaged anatomy, the centering of these structures within the field of view, and the depth and gain of the image. As the questions are view-specific and their number varies per view, we normalized the total scores between 0 and 1. To increase scoring reproducibility,^30^ videos were grouped into sets of 8 to 10 in a custom-built interface during the annotation procedure. In each group, the videos were vertically aligned, playing simultaneously. The platform enabled a rater to compare, sort, and reassess the video scores within a set to potentially rectify any initial misjudgments.

## 3 Method

**Fig. 2:**
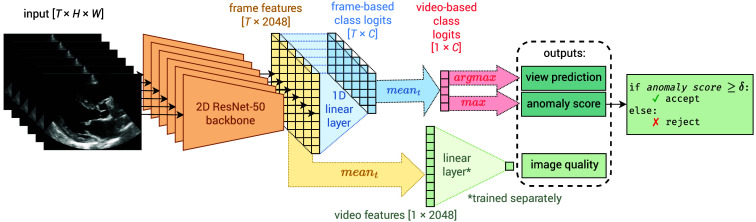
Overview of the proposed method in deployment. An echocardiography video is split into T frames, which are processed separately by a 2-dimensional ResNet-50. Its frame-based logits are averaged to give C video-based logits and the final output: A view prediction and the maximum logit, where the latter is used as an anomaly score to either accept/reject a view. In addition, the frame-based feature vectors from the ResNet-50 Backbone are averaged across frames and regressed linearly to a quality score. Note that the linear regression for quality assessment is trained separately on a small subset of videos with quality labels.

We designed an automatic method that performs view classification, unknown view recognition, and view quality assessment (see Fig. 2).

First, we train a 2D CNN (Resnet-50-v2^31^) to predict one of C=10 known view classes from an echocardiography video using cross-entropy loss. For this, we (sub-)sample a set of T still frames from the video resulting in a 3-dimensional array, X, with shape T×H×W and pixel elements $x_{tij}\in [0,1]$. During training, T is a chosen hyperparameter and the frames are sampled randomly from the video, whereas during test time, the full sequence of frames is sampled. The CNN classifier processes each frame independently, predicting a class-logit vector (y^t) for each frame. Finally, the video-based view prediction is obtained by calculating the mean of class logit vectors along the temporal dimension and taking the index of its highest value: y^=arg maxy(1T∑t=0T−1y^t).

Second, from the outputs of the trained CNN, we perform unknown view recognition. We derive an anomaly score S during test time, which is the maximum logit class activation, often abbreviated as max logit. In our context, the maximum logit is defined as S(y∈C|X)=max(1T∑t=0T−1y^t). A minimum acceptance threshold δ indicates whether a video belongs to the set C of (known) training classes or the set of unknown classes: a video with a max logit below a defined threshold will be rejected and marked as unknown.

Last, we employ the trained CNN (down to the penultimate layer) to train a quality assessment model. We employ a set of quality-rated training videos, extract the intermediate activation vectors $l_{t}$ from the CNN layer prior to the linear class mapping, and average those vectors over the frames of a video: $l=\frac{1}{T}\overset{T-1}{\underset{t=0}{\sum }}l_{t}$. We use these time-averaged feature embeddings to fit a linear regression model. Given that the feature embedding layer is high-dimensional, we impose a sparse prior on the weights: we fit a Lasso regression model, which minimizes the loss function consisting of the mean squared error and an L1-weight penalty

$$\text{Loss}=\frac{1}{2N}\overset{N}{\underset{k=1}{\sum }}{\left\|w\cdot l_{k}-y_{k}^{q}+b\right\|}_{2}^{2}+\alpha {\left\|w\right\|}_{1},$$ (1)

where $y^{q}\in [0,1]$ is the Gaudet-based^22^ quality label, w is the weights, b is the learned bias, and α is a hyperparameter to control the degree of regularization.

## 4 Evaluation

To assess the performance of our method on view classification, unknown view recognition, and quality assessment, the following metrics are used.

### 4.1 Accuracy

We define closed set accuracy and full set accuracy. Closed set accuracy is defined as the prediction accuracy on the closed dataset of known views when the unknown views (i.e., the open set) are omitted

Aclosed=|{X:y∈C∧y^=y}||{X:y∈C}|, (2)

where y is the target view class, $\hat{y}$ is the predicted class and C is the set of known classes. |·| is used to denote the cardinality (number of videos) of a set.

Full set accuracy is computed from all data (both known and unknown views) and depends on the chosen anomaly score threshold for acceptance of a video. More specifically, the full set accuracy is expressed as the sum of correctly classified known views and the correctly recognized unknown views, expressed as a fraction of the size of the full dataset

A(δ)=(|{X:y∈C∧y^=y∧S(X)≥δ}|+|{X:y∉C∧S(X)<δ}|)|{X}|, (3)

where δ is a chosen threshold on the anomaly score S(X). The δ-threshold is set to the value that maximizes A(δ) on a validation set other than the test set.

### 4.2 Receiver-Operating-Characteristic Curve (ROC-AUC)

We evaluate known-view classification performance through the area under the receiver-operating-characteristic curve (ROC-AUC). Here, we employ a one-versus-one strategy, as opposed to a one-versus-rest. Hence, the ROC-AUC is first computed among all possible pairs of known classes and then averaged. This preserves the class-distribution-agnostic properties of the ROC-AUC in a multiclass setting.^32^

Conversely, to assess the performance of unknown view recognition, we use a one-versus-rest strategy. In this case, the positives represent the accepted video samples, and the negatives represent the rejected ones. Thus, the true positive rate (TPR) and false positive rate (FPR) are defined as:

$$\mathrm{TPR}(\delta )=\frac{\left|\{X:y\in C\wedge S(X)\geq \delta \}\right|}{\left|\{X:y\in C\}\right|},$$ (4)

and

$$\mathrm{FPR}(\delta )=\frac{\left|\{X:y\notin C\wedge S(X)\geq \delta \}\right|}{\left|\{X:y\notin C\}\right|}.$$ (5)

Using these definitions, we additionally compute the balanced accuracy for unknown view recognition, which is defined as the average of TPR and specificity (1 – FPR) for a given δ.

### 4.3 Open Set Classification Rate (OSCR)

To jointly evaluate known-view classification and unknown view recognition, we compute OSCR.^14^ This equates to the true positive rate for unknown view recognition but under the extra condition that the accepted known-view predictions are correct ($\hat{y}=y$)

OSCR(δ)=|{X:y∈C∧y^=y∧S(X)≥δ}||{X:y∈C}|. (6)

We report the area under the curve of the OSCR plotted against a false positive rate.^14^

### 4.4 Spearman Correlation

To evaluate automatic view quality assessment, we assess the correlation between expert-rated and predicted quality scores using Spearman’s rank correlation coefficient, which measures how well their relation can be described by a monotonic function.

## 5 Experiments and Results

First, we describe the dataset division for the conducted experiments. Then, we describe the architecture and training of our view classification network and assess its performance on standard view classification and unknown view recognition. Finally, we evaluate our method’s ability to predict view quality.

### 5.1 Data Division

**Fig. 3:**
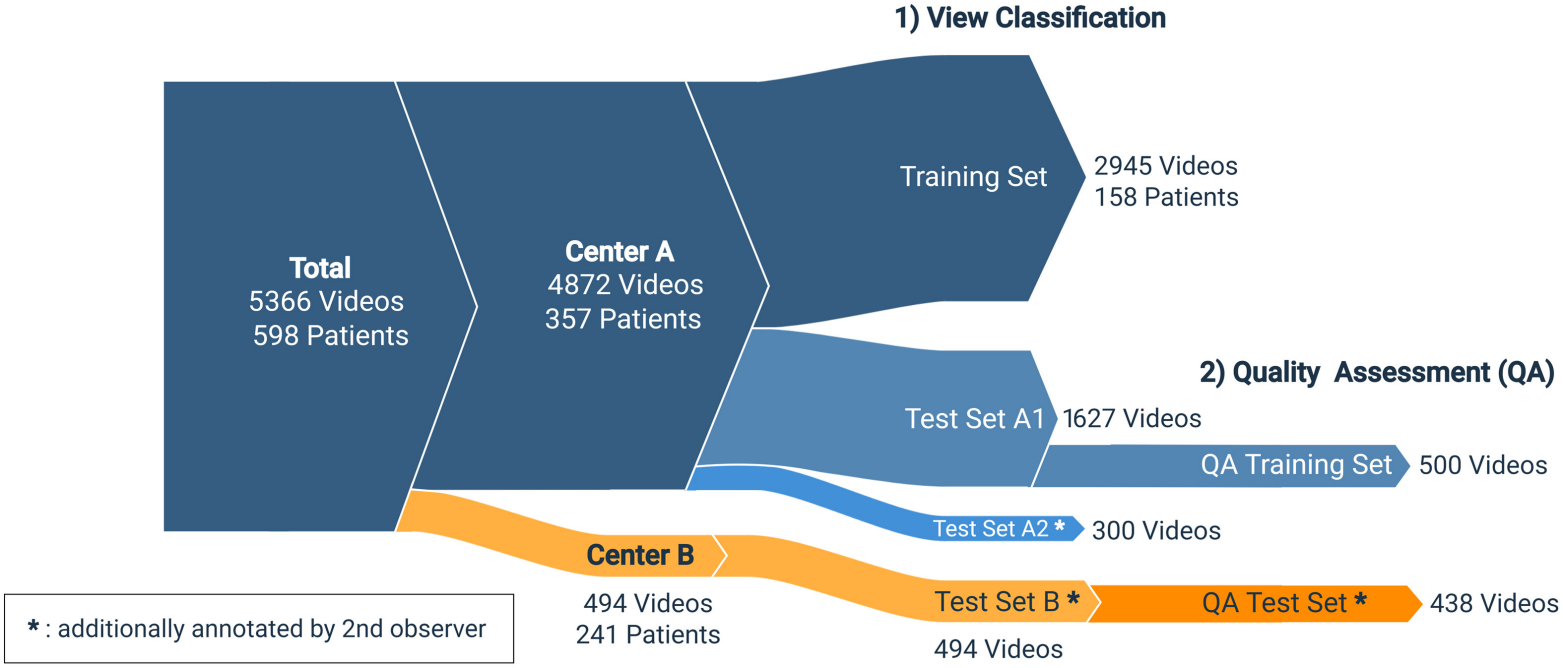
Flow diagram illustrating the dataset division for the experiments. The height of each segment is proportional to the number of videos in a set. The dataset is sourced from two centers: The data from Center A are split into a training and test set for the view classification stage of the experiments (1), after which a subset is taken to train a quality assessment model (2). Center B is part of the test set at both stages. Test sets A1 and A2 overlap on the patient level.

View classification and quality assessment were trained and evaluated successively. As illustrated in Fig. 3, for the view classification stage, the data set from Center A (357 patients), which consisted of 4872 videos, was split into a training set of 2945 videos and a test set of 1927 videos. We split the data randomly on the patient level and confirmed that the split had not introduced large distributional shifts with respect to age and sex. The training set for automatic quality assessment (QA) consists of videos sourced from the view classification test set from Center A (500 videos; 50 from each known class). Across all experiments, the included data from Center B (241 patients, 494 videos) were part of the test sets. The resulting view class distributions are detailed in Table 2. The merged sets A2+B are referred to as the interobserver test set.

**Table 2:** View-label statistics of datasets used for view classification. The number of videos is shown both as a number (n) and a percentage. The unknown views in the training set lack fine-grained labels, hence the absence of their statistics.

Dataset →	Training	Test		
		Set A1	Set A2	Set B
**Known views:**	n (%)	n (%)	n (%)	n (%)
Apical {.}				
Two-chamber	444 (15.1)	175 (10.8)	39 (13.0)	61 (12.3)
Three-chamber	277 (9.4)	145 (8.9)	26 (8.7)	43 (8.7)
Four-chamber	542 (18.4)	270 (16.6)	55 (18.3)	91 (18.4)
Five-chamber	169 (5.7)	77 (4.7)	10 (3.3)	15 (3.0)
Parasternal {.}				
Long axis	419 (14.2)	242 (14.9)	41 (13.7)	69 (14.0)
PSAX {.}				
Aortic valve	226 (7.7)	112 (6.9)	18 (6.0)	44 (8.9)
Mitral valve	221 (7.5)	106 (6.5)	18 (6.0)	35 (7.1)
Mid/apex	141 (4.8)	89 (5.5)	12 (4.0)	27 (5.5)
Subcostal {.}				
Four chamber	182 (6.2)	146 (9.0)	27 (9.0)	25 (5.1)
IVC	80 (2.7)	75 (4.6)	18 (6.0)	28 (5.7)
Total:	2701 (91.7)	1437 (88.3)	264 (88.0)	438 (88.7)
**Unknown views**:				
Novel category	—	114 (7.0)	20 (6.7)	17 (3.4)
Poor quality	—	65 (4.0)	15 (5.0)	36 (7.3)
Multiple views	—	11 (0.7)	1 (0.3)	3 (0.6)
Total:	244 (8.3)	190 (11.7)	36 (12.0)	56 (11.3)

### 5.2 View Classification and Unknown View Recognition

#### 5.2.1 Architecture and training

Prior to analysis, our video data were converted from three-channel pixel data∈[0,255] to single-channel grayscale∈[0,1] as regular (B-mode) ultrasound does not convey color information. To remove additional irrelevant information from the corners of the image, such as heart rate and ECG signal, the videos were cropped based on the video metadata. In addition, videos were resized to 224 pixels in height with a maintained image aspect ratio and center-cropped to a square image measuring 224×224  pixels.

For view classification, we used a BiTv2 architecture,^31^ with pretrained weights from Imagenet-21k. This architecture is based on Resnet-50-v2,^33^ which consists of a downsampling layer, 48 convolutional layers with an identity mapping every three layers, and a fully connected layer. We used the adjusted version of this architecture, where the batch normalization layers are replaced with a combination of group normalization^34^ and weight standardization,^35^ which allowed efficient pretraining at scale on Imagenet-21k.^31^ To process single-channel gray-scale images, we averaged the initial weights in the input layer, which normally process three-channel RGB input.

To augment training data, a cascade of random transformations was performed, each with 50% probability in the following order: rotation with the angle uniformly sampled between −25 and 25 degrees; gamma correction with gamma uniformly sampled between 0.5 and 2; resized crop with scaling parameter uniformly sampled between 0.2 and 2; elastic distortion with intensity scaling factor α set to 2 in x- and y-direction;^36^ and additive Gaussian noise with standard deviation 0.01.

Mini-batches consisted of 16 videos with eight still frames, where frames were sampled randomly from each video sequence (without replacement). Preliminary experiments showed that the number of sampled frames did not appreciably affect training performance, and eight frames per video were chosen to make optimal use of the available memory resources. To address the unbalanced class distribution in the data, the data sampler sampled each class uniformly with replacement of drawn samples. The network was trained for 300 epochs, and the learning rate was reduced by a factor of 10 after the 150th and 250th epoch. Here, we defined an epoch as the number of iterations it would take to sample all the videos of the training set without uniform sampling or replacement. This means that, during an epoch, videos from overrepresented classes were more likely to be skipped and videos from underrepresented classes were more likely to be sampled multiple times. The network was optimized using cross-entropy loss and AdamW optimizer with a learning rate of 1e−4, β equal to (0, 0.999), ϵ set to 1e−8, and weight decay set to 0.01.^37^ For testing, the minimum acceptance threshold for the anomaly score was set to the threshold that maximizes the full set accuracy on the validation set.

#### 5.2.2 Results

**Fig. 4:**
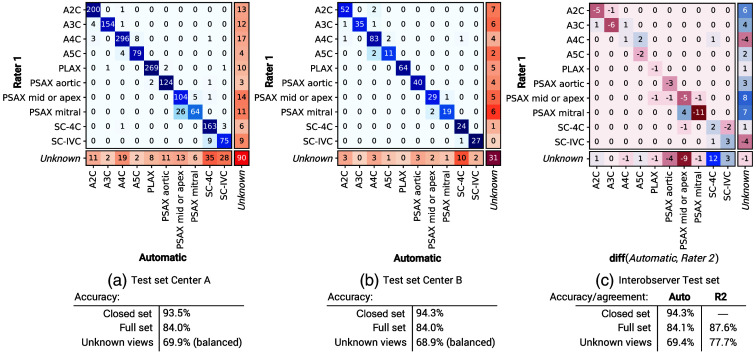
Confusion matrices for several experiments, displayed with closed set accuracy [Eq. (2)], Full set accuracy [Eq. (3)] and (balanced) unknown-view recognition accuracy (Sec. 4.2). The last row and column (shades of orange) represent unknown-view reference labels and predictions, respectively. (a) Proposed method evaluated on set A (A1+A2, Fig. 3). (b) Proposed method evaluated on set B. (c) Results on the interobserver test set (A2+B, Fig. 3).

To investigate the model’s ability to jointly perform view classification and unknown view recognition, we evaluated its performance on videos with both known and unknown views. The results are summarized in Fig. 4. On the test set from Center A [Fig. 4(a)], the model achieved 84.0% full set accuracy [Eq. (3)] and 93.5% closed set accuracy [Eq. (2)]. On the cross-vendor test set from Center B [Fig. 4(b)], the model shows no performance degradation compared with test set A, which originates from that same center as the training set.

**Fig. 5:**
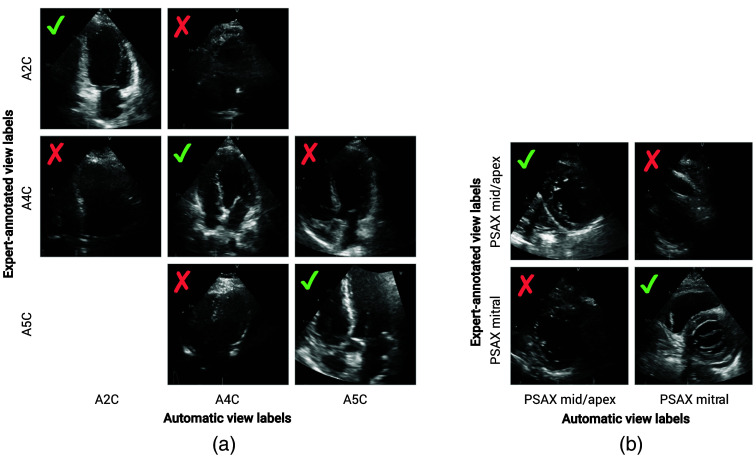
Example test images of most frequently misclassified views from the apical (a) and parasternal (b) window. Misclassified views can generally be attributed to difficult examples with poor visibility of the anatomical features that distinguish one view from another.

The most frequent disagreement of our model with the reference labels [Fig. 4(a)] can be seen among the following pair of classes: PSAX mid/apex and PSAX mitral, where disagreement was found for 31 videos in total. Other notable errors were observed with the apical four-chamber view (A4C), which was misclassified in 11 cases as either A2C or A5C. Example images from these classification results are shown in Fig. 5. As these examples suggest, most classification mistakes can be attributed to videos with poor visibility of anatomical features that distinguish view classes, such as the ascending aorta for the A5C and the mitral valve for the PSAX mitral.

With the chosen anomaly acceptance threshold, the proposed method moderately favors false acceptances over false rejections: Out of 1627 videos from test set A, we found 135 false positives and 99 false negatives. Among the false positives, there is a high prevalence of videos classified as subcostal views. Upon further inspection, we found that those views indeed belong to the broader category of subcostal views but not one of the known subcostal views from the training set. Moreover, a visual comparison of the empirical anomaly score distributions of known and unknown views revealed no notable differences between the validation and test sets.

To compare model performance with interobserver variability, we evaluated the model on the interobserver test set [Fig. 4(c)]. Although the observers agreed on the classification in 87.6% of the views, the model agreed for 84.1% of cases with rater 1, thereby approaching interobserver agreement.

Inference times for the automatic method averaged at 180 ms per video and 1.63 ms per frame on consumer-grade hardware (11th Gen Intel Core i7-11700 @ 2.5 GHz CPU, GeForce RTX 3070 GPU, and SSD data storage). As a typical echocardiography acquisition records at ∼20  ms per frame, the algorithm can be deployed in real time.

### 5.3 Quality Assessment

#### 5.3.1 Training

For quality assessment, we used the QA training set and test set as detailed in Fig. 3 and employed the trained CNN described in Sec. 6.1 (with frozen weights). We fed the videos from the QA training set to the view classifier and extracted time-averaged feature vectors from the penultimate layer. Subsequently, we used these feature vectors to train a fivefold cross-validated linear regression model with L1 regularization, using the LassoCV module from the Scikit-learn Python library. During test time, the videos of the test set were fed successively through the view classification model and the linear model to produce quality assessment predictions (Fig. 2). All predictions of the regression model were clamped between 0 and 1.

#### 5.3.2 Results

**Fig. 6:**
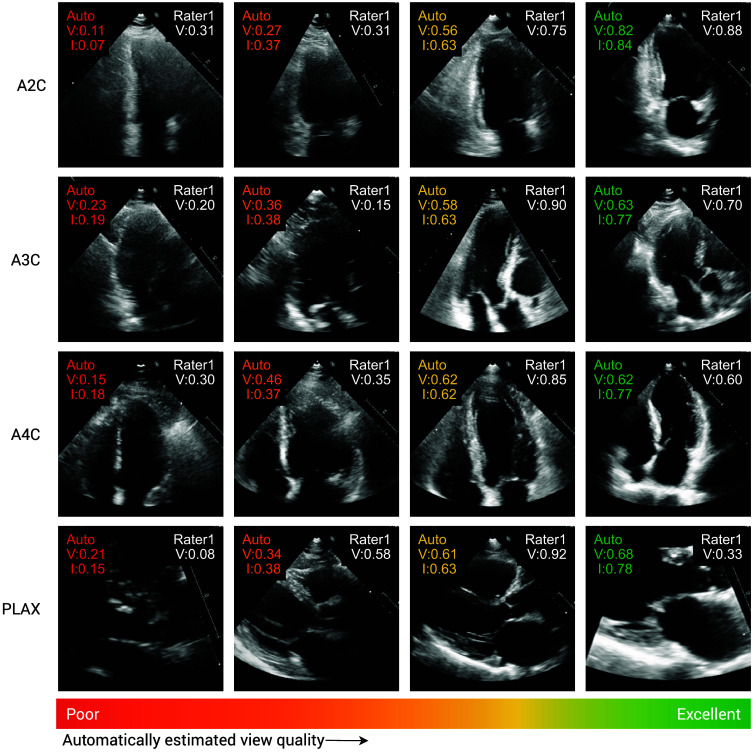
Example images from the quality assessment test set with automatic (auto) and expert view quality ratings (R1). Automatic ratings are given both as frame-level (I) and video-level (V) estimations, whereas expert ratings only exist on the video level.

**Fig. 7:**
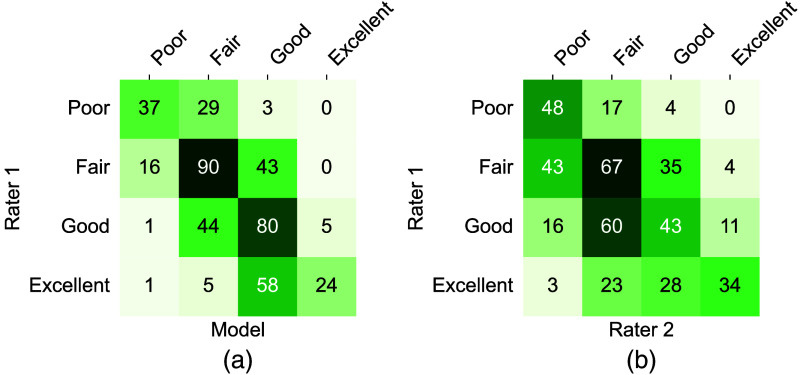
Confusion matrices of view quality assessment on test set B. (a) Model agreement with rater 1 (52.7%). (b) Interobserver agreement (44.0%). Normalized quality scores were binned according to the following intervals: poor [0, 0.25], fair (0.25, 0.5], good (0.5, 0.75], and excellent (0.75, 1].

The achieved results are summarized in Table 3 and Figs. 6 and 7. Figure 6 shows test examples ranked by automatic quality scores in ascending order, where a clear positive trend in view quality can be observed from left to right. The automatic ranking disagrees mostly with the expert ratings in the last column: two disagreements between the good and excellent categories, and a zoomed PLAX view, which was rated only as fair by the expert. As Fig. 7 shows, the model agrees in 52.7% of cases with rater 1, whereas in 96.6% of cases, the agreement between the method and rater 1 is at most one category off. This surpasses the inter-rater agreement levels, which are only 44.0% and 88.6%, respectively. Spearman’s Rank correlation coefficients (Table 3) reveal that the quality scores predicted by our model are closely aligned with the assessments of rater 1, which labeled the training data. On average, this alignment surpasses the correlation between rater 1 and rater 2. Only A3C and PLAX videos show superior agreement between raters.

**Table 3:** Inter-rater and model-rater Spearman correlation on video quality assessment (QA)—evaluated on the QA test set (Fig. 3). R1 and R2 stand for raters 1 and 2, respectively. The QA training set was annotated by rater 1. The best result among the three comparisons is displayed in bold font.

Comparison:	Spearman’s Rank Corr. Coef. (↑)		
	R1/R2	R1/model	R2/model
A2C	0.629	**0.747**	0.536
A3C	**0.864**	0.587	0.706
A4C	0.668	**0.733**	0.606
A5C	0.665	**0.798**	0.542
PLAX	**0.773**	0.703	0.522
PSAX aortic	0.338	**0.706**	0.485
PSAX mitral	0.708	**0.785**	0.539
PSAX mid or apex	0.634	**0.734**	0.517
SC-4C	0.646	**0.762**	0.510
SC-IVC	0.229	**0.504**	0.091
Mean ± std	0.62±0.18	0.71±0.09	0.51±0.15

## 6 Additional Experiments

### 6.1 Training Classifier with Unknown Views as Outlier Category

In our earlier experiments, the available unknown views were included in the test set but excluded from the training set. This simulates a realistic clinical scenario, where the model could be exposed to unknown unknowns. However, to assess the benefits of using the available unknown views for training, we conducted an experiment using outlier exposure.^38^ For this, we included the available unknown views in the training set and added an extra class to the model backbone to represent these videos. This effectively turns those classes into known unknowns, which makes this experiment deviate from our original focus on detecting unknown unknowns.

For training, all reference labels of outlier videos were collapsed to a single outlier class without further specification of the fine-grained (outlier) label. The network architecture was adjusted by including an extra (scalar) output logit, appended to the logit vector representing the in-distribution class activations. For this experiment, we maintained class-balanced sampling, treating the outlier class as one of the in-distribution classes.

For testing, the in-distribution prediction of a video was inferred to be the largest activation of the known class logits. To compute the anomaly score, we experimented with the two following configurations: (a) In line with the proposed method, we computed the maximum logit activation over the known classes, now explicitly ignoring the outlier class activation,^20^ and (b) we sum the Softmax probabilities of the known classes^38^ (i.e., summed Softmax).

**Fig. 8:**
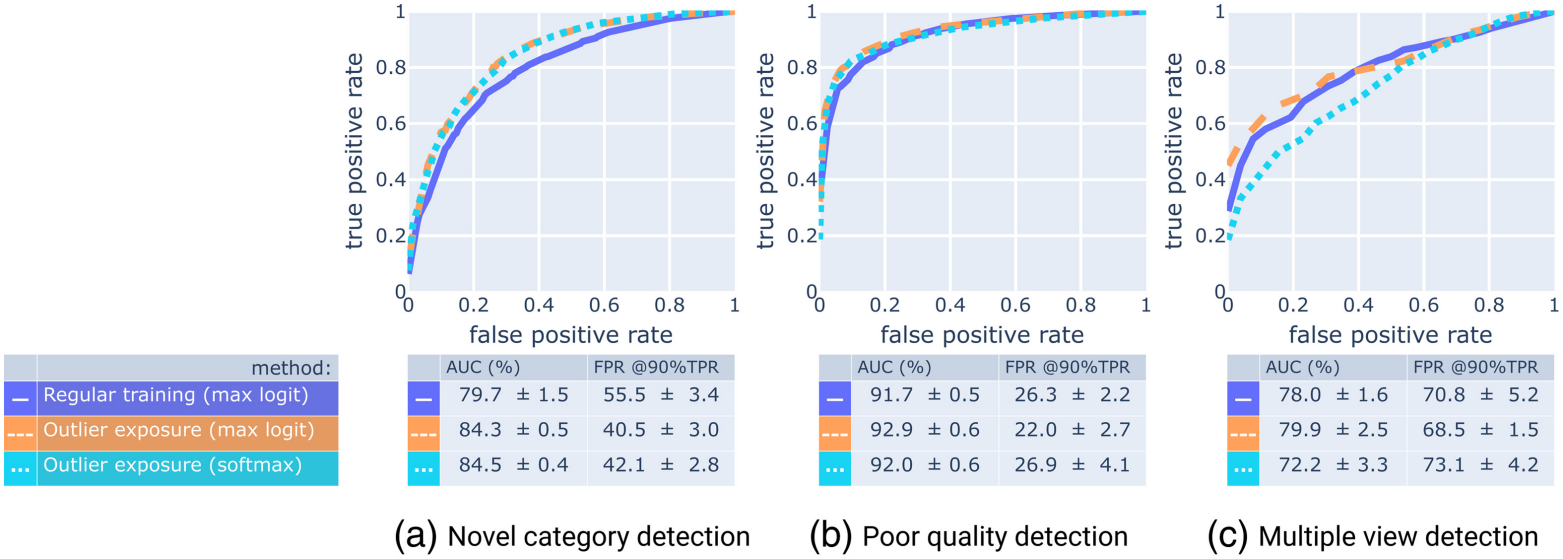
Cross-validated performance of training with outlier exposure in comparison with regular training on three different subtasks (a)–(c) of unknown view detection on the full test set (A1+A2+B, Fig. 3). For each subtask, we generate the ROC curves by removing the samples of the other two subcategories from the test set. To obtain the curves as displayed, we average the (interpolated) true positive rates across the validation folds.

To allow a meaningful comparison between approaches, we trained both the regular and outlier-exposed model using fivefold cross-validation (see Sec. 9.1). The results are summarized in Table 4 and Fig. 8. Although outlier exposure did not improve closed set accuracy and known-view ROC-AUC, Table 4 shows that outlier exposure led to improvements in full set accuracy, open set classification rate (OSCR), and unknown-view detection ROC-AUC. As Fig. 8 illustrates, the enhanced metrics are due to the improved detection of novel categories. Finally, the max logit activation outperforms the Softmax-based anomaly score, most notably on multiple view detection.

**Table 4:** Comparison of the proposed method with regular training (Sec. 5.2.1) and training with outlier exposure (Sec. 6.1). Evaluation metrics include closed set accuracy [Eq. (2)], full set accuracy [Eq. (3)], open set classification rate AUC (OSCR-AUC) [Eqs. (5), (6)], known view classification ROC-AUC, and unknown-view recognition ROC-AUC [Eqs. (4), (5)]. Results (mean (%) ± standard deviation) are obtained through training with fivefold cross-validation and testing on the full test set. Experiment with the greatest mean of row is displayed in bold font.

	Regular training (max logit)	Outlier exposure (max logit)	Outlier exposure (Softmax)
Closed set accuracy [Eq. (2)]	93.2±0.80	93.5±0.28	93.5±0.28
Full set accuracy [Eq. (3)]	84.9±0.67	85.8±0.44	85.1±0.30
OSCR-AUC [Eqs. (5), (6)]	78.9±1.68	82.9±0.35	80.2±0.56
ROC-AUC known views	99.4±0.06	99.1±0.04	99.1±0.04
ROC-AUC unknown views [Eqs. (4), (5)]	82.7±1.36	86.9±0.24	85.2±0.35

### 6.2 Evaluation of Public Datasets

To allow comparisons with our work, we evaluate our approach on two publicly available datasets. The first dataset, known as the CAMUS dataset,^27^ contains echocardiographic videos of apical two- and four-chamber views. The CAMUS dataset was originally developed to enable automatic left ventricle segmentation. The second dataset, known as EchoNet-LVH,^28^ consists primarily of PLAX views and was developed to enable automatic assessment of left-ventricle wall thickness.

We repurpose these datasets to test whether our model correctly identifies the views contained within them. For this, we employ the trained model from a randomly selected validation fold described in Sec. 6.1. From the public datasets, we utilized the full set as a test set and applied the same preprocessing steps, as described in Sec. 5.2.1. The results are summarized in Table 5. The proposed method achieved a view classification accuracy of 93.0% on CAMUS and 91.6% on EchoNet-LVH.

**Table 5:** Classification results on the CAMUS and EchoNet-LVH datasets. The classification accuracy amounts to 93.0% on CAMUS (A2C and A4C) and 91.6% on EchoNet-LVH (PLAX). Numbers in bold font indicate correct classifications.

		Reference		
		A2C	A4C	PLAX
Predicted	A2C	**478**	18	28
	A3C	8	0	183
	A4C	10	**452**	30
	A5C	1	30	6
	PLAX	0	0	**10,987**
	PSAX aortic	0	0	20
	PSAX mid/apex	0	0	35
	PSAX mitral	1	0	10
	SC-4C	0	0	5
	SC-IVC	0	0	100
	Unknown	2	0	593

**Fig. 9:**
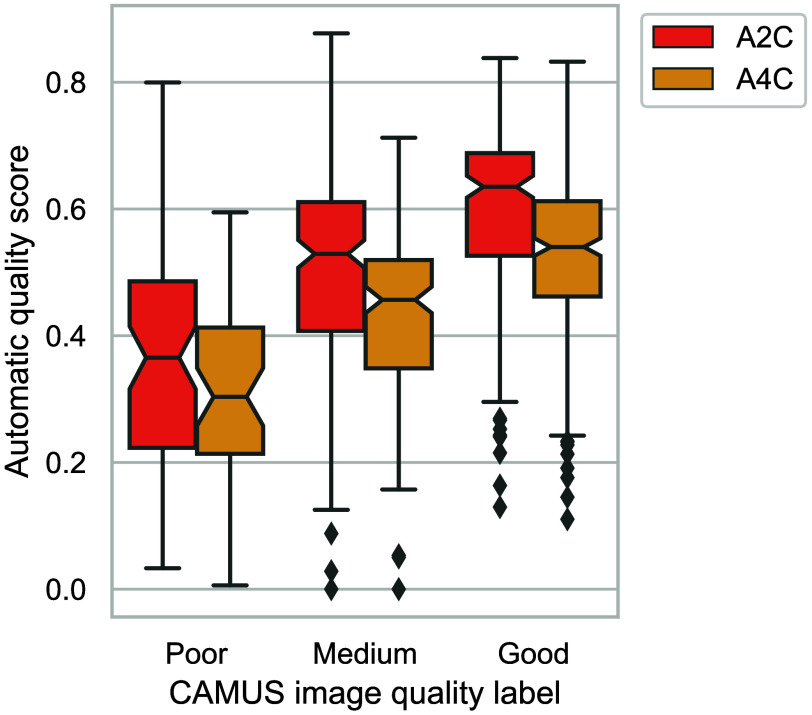
Automatically inferred view quality scores from the CAMUS dataset.^27^ The dataset provided both view and image quality labels as metadata to their videos.

Furthermore, because the CAMUS dataset includes image quality labels, we used these to evaluate our automatic method for view quality assessment. The results are summarized in Fig. 9 as box-and-whisker plots grouped by reference category (poor, medium, or good). The results demonstrate a clear positive correlation between the estimated and reference quality.

**Fig. 10:**
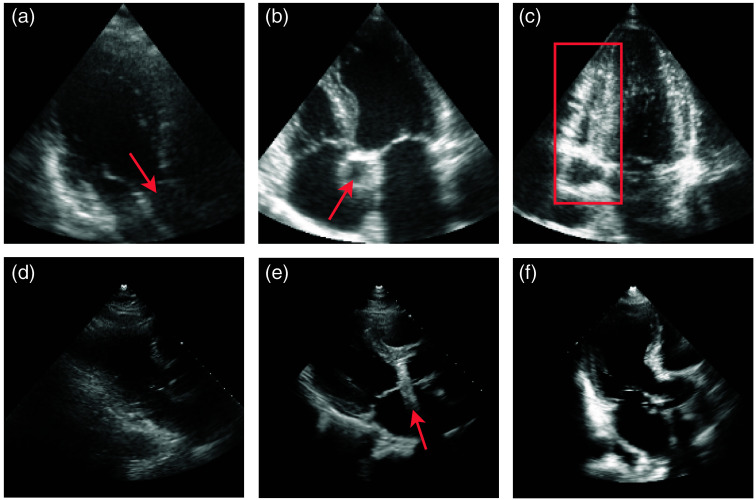
Explainable misclassification examples from CAMUS^27^ (a)–(c) and EchoNet-LVH^28^ (d)–(e). (a) Apical three-chamber view with two-chamber reference label (arrow: outflow tract). (b) Apical five-chamber view with four-chamber reference label (arrow: ascending aorta). (c) Poorly optimized apical four-chamber view (right, bounding box: right heart). (d) and (e) PLAX view inferred as unknown by proposed method (arrow: imaging artifact). (f) Apical three-chamber view with PLAX reference label.

Through the visual inspection of the misclassified views in the CAMUS dataset, we found that several videos with A2C and A4C reference labels would be more accurately described as A3C and A5C views, respectively. Two such examples are shown in Figs. 10(a) and 10(b). This finding aligns with the original dataset descriptors,^27^ which acknowledge the inclusion of A5C views within the A4C category when a true A4C view was unavailable. Our model correctly identified a significant proportion of these cases as true A5C or A3C views: 20 out of 30 cases that our model identified as five-chamber views were true A5C views. Similarly, all eight cases that our model identified as A3C views were true A3C views. In addition, misclassifications between A2C and A4C consisted entirely of edge cases or cases with poor visibility of the target anatomy.

Similar challenges were encountered with the EchoNet-LVH dataset, particularly when affected by poor image quality or artifacts [see Figs. 10(d) and 10(e)]. In some cases, the model misclassified PLAX views as one of the other known view classes, but it predominantly misidentified PLAX views as unknown. Visual inspection revealed that the PLAX views that our model classified as A3C views showed a version of the PLAX view that was rotated toward the apex, therefore resembling an A3C view. About 25% of these disagreements even showed true A3C views [Fig. 10(f)].

## 7 Discussion

We have presented an automated method for echocardiography view classification. Unlike previous works, our method also recognizes unknown views, which are common in exams, and rates view quality, which is important for downstream analysis. For these, we used a CNN that was supervised by view labels and a linear mapping of CNN features to assess view quality. These CNN features thereby allow us to automate view quality assessment using a relatively modest set of view quality training labels, which are generally expensive to acquire.

Similar to Howard et al.’s^9^ view classification method, our method approaches expert-level performance. Direct comparison of our method with previous methods is, however, challenging due to a lack of publicly shared code and data. To allow future comparisons with our method, we performed an evaluation of publicly available echocardiography data. Further improvements of our method can be made in the distinction of classes with strong visual similarity to one another, especially when the image quality is suboptimal. For example, the model sometimes mistakes the PSAX view at the mitral valve level for the PSAX view at the mid-level. The distinction between these views heavily relies on the visibility of the mitral valve, which is hardly visible on a still image if the image quality is poor. However, we would like to note that the clinical benefit of these improvements could be limited as many of these misclassified videos are characterized by insufficient diagnostic quality. A more impactful improvement would be to close the gap in performance between experts and our model for the task of unknown view recognition. As shown in Fig. 4, a limitation of the current method is the relatively low balanced accuracy compared with the second observer. Future work could potentially improve on this by shifting from a post-hoc derived anomaly score to a representation learning approach such as prototype learning or reciprocal point learning.^19^^,^^39^

The three subcategories of unknown views have shown to be a relevant stratification of anomaly detection in echocardiography as each subcategory showed different responses to max-logit-based detection. Among the subcategories, the proposed method is most successful at detecting unrecognizable videos with poor image quality. As videos with poor image quality lack the defining structures and anatomical features of a view, it is hardly surprising that a view classifier returns low-class activations for these videos. The results show that our method struggles to detect novel categories, likely misclassifying them due to the presence of familiar features. The method most notably fails with novel subcostal views, such as the subcostal short-axis view, possibly due to shared image features with the known subcostal views. Another limitation is that the method does not differentiate subcategories of unknown views, which could be useful in limited cases where a video fluctuating among views shows diagnostic-quality anatomy and should not be categorized as unusable.

We have shown that novel category detection can be improved by training with outlier exposure. However, model performance may degrade when exposed to novel types of outliers. The unexposed model may be a plausible lower bound on this performance degradation. Outlier echocardiograms are nevertheless an inevitable byproduct of data acquisition, and we have shown that utilizing these echocardiograms will result in optimal performance with the available resources, thereby likely improving clinical utility.

The Softmax-based anomaly score performed comparably to the maximum logit on novel category detection and poor-quality detection but more often failed to detect when the view fluctuated. This means that taking the sum of Softmax probabilities (across the known classes) yields fewer anomalous values for multiple-view sequences than taking the max logit. This could be because videos with multiple views typically alternate between two known views, causing Softmax activations to sum to a normal (non-anomalous) value.

Choosing the threshold δ for the anomaly score significantly impacts the method’s performance. We determined the optimal threshold based on the maximum achievable full set accuracy from the validation set. However, this metric is sensitive to the frequency of unknown views relative to known views, which is problematic if this ratio is unpredictable. To address this, an alternative approach is to use the validation set to determine the threshold based on the average of OSCR and unknown view recognition specificity. This metric remains invariant to the relative frequency of unknown views and can achieve a maximum value at a threshold within its domain that is not at the extremes of all possible δ values.

The key limitation of our proposed echocardiography view classifier is the incomplete list of known views for classification. Although Howard et al.^9^ and Zhang et al.^7^ recognized 14 and 22 views, respectively, we incorporated 10, as those were the available views in our centers. Although the study of Zhang et al.^7^ remains the most extensive to date, no study currently includes every possible view, which necessitates the open set recognition approach. Future enhancements could involve multi-center data and broader inclusion criteria to allow a more comprehensive set of routine views.

Our view quality assessment method predicts quality scores within the range of inter-rater variability using a modest training set of 50 videos per class. This is enabled by view classification features, which we have shown are useful toward predicting view quality. View quality assessment is challenging due to significant intra- and inter-rater variability. Similar to Liao et al.,^21^ who found only 55% intra-rater agreement, we found 44.0% inter-rater agreement. Although our model yielded a 52.7% agreement with rater 1, our method did not achieve comparable agreement to rater 2, as is demonstrated by inferior Spearman correlation coefficients. To enhance this method, we suggest using multiple raters to annotate the training set, which gives an empirical distribution of quality labels. This allows for the implementation of the multi-label soft-labeling approach proposed by Liao et al.^21^ This combination could provide insight into when a quality estimation is useful, gauged by an uncertainty estimate, while leveraging the efficiency of our pre-trained view classifier backbone that has already learned the essential features. As the inter-rater agreement strongly differs per view, it is plausible that the view quality reference standards for some views are sub-optimally defined. Thus, future work could investigate these reference standards with the goal of minimizing inter-rater variability and improving clinical usefulness for downstream analyses.

The current work allows quick sorting and querying of echocardiographic studies to improve the diagnostic workflow. The automatic view labeling makes the studies queryable with a search term for the desired view, and the automatic quality scores can automatically provide the best acquisition among the list of matches. In addition, the predicted quality of acquisition is important for automating downstream analyses such as ejection fraction and strain estimation, as it may automatically filter out views that are unfit for these purposes. Given this, the current method may aid in reducing the level of skill required for acquiring echocardiograms, thereby adding value to point-of-care ultrasound devices in general practice care and medically under-served areas.

## 8 Conclusion

We have presented a CNN-based method for real-time automatic 2D-echocardiography view classification with unknown view recognition and automatic assessment of view quality. The proposed method achieved an accuracy of 84.9% for the joint objective of routine view classification and unknown view recognition, whereas a second observer reached an accuracy of 87.6%. For view quality assessment, Spearman’s rank correlation coefficient between our method and the reference was 0.71, whereas 0.62 for a second observer. The results indicate that the method enables fully automatic selection of the highest quality target views for manual or automatic downstream analysis.

## 9 Appendix

### 9.1 Comparison of Anomaly Scores

To demonstrate the benefit of using the max logit as the proposed score for anomaly detection, we compared this score with other frequently used anomaly scores. For this evaluation, we trained the model as described in Sec. 5.2.1, now using fivefold cross-validation on our training set to allow a statistically meaningful comparison among anomaly scores. Then, we computed the following post-hoc anomaly scores for each video in the test set, in addition to the max logit

1. Maximum Softmax probability: the highest estimated probability among classes. This score is computed by normalizing the logits with the Softmax function and then taking the maximum value among the classes. Anomalous videos should correspond to low maximum Softmax values.^16^
2. L1-norm of feature layer (L1): the mean of feature activations in the penultimate layer of the network.^15^ We averaged the activations across the time dimension before taking the mean activation of the 2048-dimensional feature layer. Anomalous videos should be represented by low L1 norms.
3. Entropy of feature layer: the entropy of normalized activations in the penultimate layer of a network. To compute this score for a single video, we averaged the feature vectors across time, then we normalized the vector to sum to 1, and finally, we computed the entropy. We negated this score such that low scores correspond to anomalous videos.
4. Relative frequency: occurrence of the most frequently predicted view on a frame-by-frame basis, computed as a fraction of the total number of frames in a video. Anomalous videos should be associated with lower relative frequencies.

Finally, we used ROC curves to compare the anomaly scores in their ability to discriminate known views from three subcategories of unknown views: (a) videos from novel categories, (b) videos with poor image quality, and (c) videos showing multiple views over time.

**Fig. 11:**
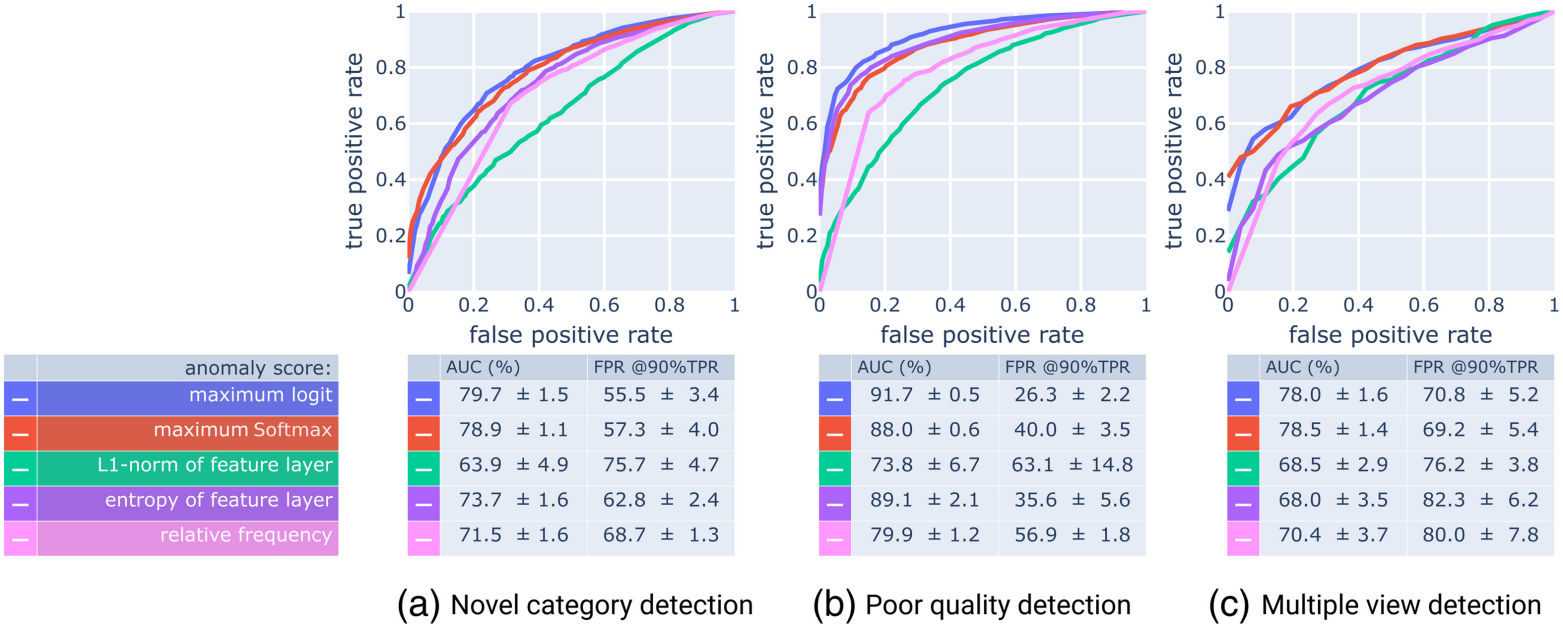
Cross-validated performance of four different anomaly scores on three different subtasks (a)–(c) of unknown view detection on the full test set (A1+A2+B, Fig. 3). For each subtask, we generate the ROC curves by removing the samples of the other two subcategories from the test set. To obtain the curves as displayed, we average the (interpolated) true positive rates across the validation folds.

These results are summarized in Fig. 11, which shows that the max logit activation is the best-performing anomaly score among the ones that we tested, closely followed by the maximum Softmax probability, and feature layer entropy. This is consistent with the findings of Dietterich and Guyer^20^ who listed the max logit as the most successful familiarity-based anomaly score.

### 9.2 2D versus 3D Convolutional Architecture

To investigate whether a spatiotemporal 3D CNN leads to improved performance over the 2D CNN in the proposed method (Resnet-50, BiTv2), we trained a 3D variant of Resnet (X3D-M). This model adheres to the architecture design of a 2D Resnet-50 but with 3D instead of 2D convolutional kernels, higher parameter efficiency, and pretrained weights from the kinetics-400 video action recognition dataset,^40^ instead of Imagenet-21k. We modified our training and inference protocol to fit a 3D architecture: During training, we down-sampled a video sequence by an integer factor M that resulted in a frame rate as close to 16 fps as possible and selected a random 16-frame sequence. For testing, we divided the entire video into successive 16-frame sequences at the same fps, with each sequence overlapping the preceding and following sequences by eight frames. The final output for each test video was computed by averaging the outputs of all its sequences. The remaining training and testing conditions remained identical to the 2D model.

As with previously tested approaches, the 3D model was trained using fivefold cross-validation. The results are summarized in Table 6. Despite similar classification ROC-AUC, the 3D model critically underperforms on metrics for unknown view recognition compared with the 2D model, namely, unknown view recognition ROC-AUC and open-set-classification-rate AUC.

**Table 6:** Comparison of the proposed 2D CNN with a 3D CNN. Evaluation metrics include closed set accuracy [Eq. (2)], full set accuracy [Eq. (3)], open set classification rate AUC (OSCR-AUC) [Eqs. (5), (6)], known view classification ROC-AUC, and unknown-view recognition ROC-AUC [Eqs. (4), (5)]. Results (mean (%) ± standard deviation) are obtained through training with fivefold cross-validation and testing on the full test set. The max logit is used as the anomaly score. Experiment with greatest mean of row is displayed in bold font.

	2D CNN	3D CNN
Closed set accuracy [Eq. (2)]	93.2±0.80	91.3±0.36
Full set accuracy [Eq. (3)]	84.9±0.67	83.0±0.36
OSCR-AUC [Eqs. (5), (6)]	78.9±1.68	73.3±1.14
ROC-AUC known views	99.4±0.06	99.2±0.12
ROC-AUC unknown views [Eqs. (4), (5)]	82.7±1.36	78.1±0.99

We hypothesized that by utilizing temporal information, a 3D CNN could improve upon the 2D approach. A 3D approach would be able to identify distinctly moving structures in similar views such as the PSAX views, where only one of them shows the (moving) mitral valve. However, the results from the tested 3D CNN did not show improved discrimination. Possible improvements for the current 3D implementation could include a longer temporal window in conjunction with a higher frame sampling rate to capture more fine-grained spatiotemporal information.

## Data Availability

The code used to generate the results is not available due to funding source restrictions. The data are not publicly available due to privacy concerns. For inquiries, please contact the corresponding author at g.e.jansen@amsterdamumc.nl.

## References

[1] Schuuring M. J. et al., “Routine echocardiography and artificial intelligence solutions,” Front. Cardiovasc. Med. 8, 648877 (2021). 10.3389/fcvm.2021.648877 33708808 PMC7940184

[2] Gahungu N. et al., “Current challenges and recent updates in artificial intelligence and echocardiography,” Curr. Cardiovasc. Imaging Rep. 13, 1–12 (2020). 10.1007/s12410-020-9529-x

[3] Penatti O. A. B. et al., “Mid-level image representations for real-time heart view plane classification of echocardiograms,” Comput. Biol. Med. 66, 66–81 (2015). CBMDAW 0010-4825 10.1016/j.compbiomed.2015.08.004 26386547

[4] Ebadollahi S. Chang S. F. Wu H., “Automatic view recognition in echocardiogram videos using parts-based representation,” in Proc. IEEE Comput. Soc. Conf. Comput. Vision and Pattern Recognit., Vol. 2 (2004). 10.1109/CVPR.2004.1315137

[5] Gao X. et al., “A fused deep learning architecture for viewpoint classification of echocardiography,” Inf. Fusion 36, 103–113 (2017). 10.1016/j.inffus.2016.11.007

[6] Madani A. et al., “Fast and accurate view classification of echocardiograms using deep learning,” NPJ Digital Med. 1(1), 6 (2018). 10.1038/s41746-017-0013-1 PMC6395045 30828647

[7] Zhang J. et al., “Fully automated echocardiogram interpretation in clinical practice,” Circulation 138, 1623–1635 (2018). CIRCAZ 0009-7322 10.1161/CIRCULATIONAHA.118.034338 30354459 PMC6200386

[8] Østvik A. et al., “Real-time standard view classification in transthoracic echocardiography using convolutional neural networks,” Ultrasound Med. Biol. 45, 374–384 (2019). USMBA3 0301-5629 10.1016/j.ultrasmedbio.2018.07.024 30470606

[9] Howard J. P. et al., “Improving ultrasound video classification: an evaluation of novel deep learning methods in echocardiography,” J. Med. Artif. Intell. 3, 4 (2020). 10.21037/jmai.2019.10.03 32226937 PMC7100611

[10] Vaseli H. et al., “Designing lightweight deep learning models for echocardiography view classification,” Proc. SPIE 10951, 109510F (2019). PSISDG 0277-786X 10.1117/12.2512913

[11] Azarmehr N. et al., “Neural architecture search of echocardiography view classifiers,” J. Med. Imaging 8, 034002 (2021). JMEIET 0920-5497 10.1117/1.JMI.8.3.034002 PMC8217960 34179218

[12] Huang G. et al., “Densely connected convolutional networks,” in Proc. IEEE Comput. Soc. Conf. Comput. Vision and Pattern Recognit., pp. 4700–4708 (2017).

[13] Geng C. Huang S. J. Chen S., “Recent advances in open set recognition: a survey,” IEEE Trans. Pattern Anal. Mach. Intell. 43, 3614–3631 (2021). ITPIDJ 0162-8828 10.1109/TPAMI.2020.2981604 32191881

[14] Dhamija A. R. Günther M. Boult T., “Reducing network agnostophobia,” in Adv. Neural Inf. Process. Syst. 31, Curran Associates, Inc. (2018).

[15] Vaze S. et al., “Open-set recognition: a good closed-set classifier is all you need,” in Int. Conf. Learn. Represent. (2022).

[16] Hendrycks D. Gimpel K., “A baseline for detecting misclassified and out-of-distribution examples in neural networks,” in Int. Conf. Learn. Represent. (2017).

[17] Lee K. et al., “A simple unified framework for detecting out-of-distribution samples and adversarial attacks,” in Adv. Neural Inf. Process. Syst. 31, Curran Associates, Inc. (2018).

[18] Bendale A. Boult T. E., “Towards open set deep networks,” in Proc. IEEE Comput. Soc. Conf. Comput. Vision and Pattern Recognit., 2016-December, pp. 1563–1572 (2016).

[19] Chen G. et al., “Adversarial reciprocal points learning for open set recognition,” IEEE Trans. Pattern Anal. Mach. Intell. 44, 8065–8081 (2021). ITPIDJ 0162-8828 10.1109/TPAMI.2021.3106743 34428133

[20] Dietterich T. G. Guyer A., “The familiarity hypothesis: explaining the behavior of deep open set methods,” Pattern Recognit. 132, 108931 (2022). PTNRA8 0031-3203 10.1016/j.patcog.2022.108931

[21] Liao Z. et al., “On modelling label uncertainty in deep neural networks: automatic estimation of intra-observer variability in 2D echocardiography quality assessment,” IEEE Trans. Med. Imaging 39, 1868–1883 (2020). ITMID4 0278-0062 10.1109/TMI.2019.2959209 31841401

[22] Gaudet J. et al., “Focused critical care echocardiography: development and evaluation of an image acquisition assessment tool,” Crit. Care Med. 44, e329–e335 (2016). CCMDC7 0090-3493 10.1097/CCM.0000000000001620 26825858

[23] Abdi A. H. et al., “Automatic quality assessment of echocardiograms using convolutional neural networks: feasibility on the apical four-chamber view,” IEEE Trans. Med. Imaging 36, 1221–1230 (2017). ITMID4 0278-0062 10.1109/TMI.2017.2690836 28391191

[24] Abdi A. H. et al., “Quality assessment of echocardiographic cine using recurrent neural networks: feasibility on five standard view planes,” Lect. Notes Comput. Sci. 10435, 302–310 (2017). LNCSD9 0302-9743 10.1007/978-3-319-66179-7_35

[25] Labs R. B. Zolgharni M. Loo J. P., “Echocardiographic image quality assessment using deep neural networks,” Lect. Notes Comput. Sci. 12722, 488–502 (2021). LNCSD9 0302-10.1007/978-3-030-80432-9_36

[26] Zamzmi G. et al., “Real-time echocardiography image analysis and quantification of cardiac indices,” Med. Image Anal. 80, 102438 (2022). 10.1016/j.media.2022.102438 35868819 PMC9310146

[27] Leclerc S. et al., “Deep learning for segmentation using an open large-scale dataset in 2D echocardiography,” IEEE Trans. Med. Imaging 38, 2198–2210 (2019). ITMID4 0278-0062 10.1109/TMI.2019.2900516 30802851

[28] Duffy G. et al., “High-throughput precision phenotyping of left ventricular hypertrophy with cardiovascular deep learning,” JAMA Cardiol. 7, 386 (2022). 10.1001/jamacardio.2021.6059 35195663 PMC9008505

[29] Molenaar M. A. et al., “The impact of valvular heart disease in patients with chronic coronary syndrome,” Front. Cardiovasc. Med. 10, 1211322 (2023). 10.3389/fcvm.2023.1211322 37547247 PMC10401435

[30] Larson E. C. Chandler D. M., “Most apparent distortion: full-reference image quality assessment and the role of strategy,” J. Electron. Imaging 19, 011006 (2010). JEIME5 1017-9909 10.1117/1.3267105

[31] Kolesnikov A. et al., “Big transfer (bit): general visual representation learning,” Lect. Notes Comput. Sci. 12350, 491–507 (2020). 10.1007/978-3-030-58558-7_29

[32] Hand D. J. Till R. J., “A simple generalisation of the area under the ROC curve for multiple class classification problems,” Mach. Learn. 45, 171–186 (2001). MALEEZ 0885-6125 10.1023/A:1010920819831

[33] He K. et al., “Identity mappings in deep residual networks,” Lect. Notes Comput. Sci. 9908, 630–645 (2016). 10.1007/978-3-319-46493-0_38

[34] Wu Y. He K., “Group normalization,” in Proc. Eur. Conf. Comput. Vision (ECCV) (2018).

[35] Qiao S. et al., “Micro-batch training with batch-channel normalization and weight standardization,” arXiv:1903.10520 (2019).

[36] Simard P. Y. Steinkraus D. Platt J. C., “Best practices for convolutional neural networks applied to visual document analysis,” in Seventh Int. Conf. Doc. Anal. and Recognit., 2003. Proc., pp. 958–963 (2003).

[37] Loshchilov I. Hutter F., “Decoupled weight decay regularization,” in Int. Conf. Learn. Represent. (2019).

[38] Fort S. Ren J. Lakshminarayanan B., “Exploring the limits of out-of-distribution detection,” in Adv. Neural Inf. Process. Syst. 34, pp. 7068–7081 (2021).

[39] Chen T. et al., “A simple framework for contrastive learning of visual representations,” in 37th Int. Conf. Mach. Learn., ICML (2020).

[40] Feichtenhofer C., “X3D: expanding architectures for efficient video recognition,” in Proc. IEEE/CVF Conf. Comput. Vision and Pattern Recognit. (CVPR) (2020). 10.1109/CVPR42600.2020.00028

